# Immunomodulatory biomaterials on chemokine signaling in wound healing

**DOI:** 10.3389/fphar.2023.1084948

**Published:** 2023-04-21

**Authors:** Anisha Apte, Kenneth W. Liechty, Carlos Zgheib

**Affiliations:** Laboratory for Fetal and Regenerative Biology, Department of Surgery, University of Arizona College of Medicine, Tucson, AZ, United States

**Keywords:** wound healing, chemokines, biomaterials, immunomodulation, chronic wounds, diabetic wounds

## Abstract

Normal wound healing occurs through a careful orchestration of cytokine and chemokine signaling in response to injury. Chemokines are a small family of chemotactic cytokines that are secreted by immune cells in response to injury and are primarily responsible for recruiting appropriate immune cell types to injured tissue at the appropriate time. Dysregulation of chemokine signaling is suspected to contribute to delayed wound healing and chronic wounds in diseased states. Various biomaterials are being used in the development of new therapeutics for wound healing and our understanding of their effects on chemokine signaling is limited. It has been shown that modifications to the physiochemical properties of biomaterials can affect the body’s immune reaction. Studying these effects on chemokine expression by various tissues and cell type can help us develop novel biomaterial therapies. In this review, we summarize the current research available on both natural and synthetic biomaterials and their effects on chemokine signaling in wound healing. In our investigation, we conclude that our knowledge of chemokines is still limited and that many in fact share both pro-inflammatory and anti-inflammatory properties. The predominance of either a pro-inflammatory or anti-inflammatory profile is mostly likely dependent on timing after injury and exposure to the biomaterial. More research is needed to better understand the interaction and contribution of biomaterials to chemokine activity in wound healing and their immunomodulatory effects.

## Introduction

Chemokines are a family of chemotactic cytokines that are primarily responsible for recruiting the appropriate immune cell type to injured tissue at the appropriate time (Satish, 2015). Chemokines are secreted by both innate and adaptive immune cells in response to injury (Sokol and Luster, 2015). There are about 50 chemokines that have been identified thus far, with four subgroups: C-motif, ligand (CL), CC-motif ligand (CCL) CXC-motif ligand (CXCL) and CXC3-motif ligand (CXC3L). Among these, the CCL and CXCL chemokines are the most common (Satish, 2015). Our knowledge about the specific roles of each chemokine in wound healing is limited, but we know that their regulation is essential to facilitating timely progression between stages of wound healing (Childs and Murthy, 2017). Dysregulated expression of chemokines may lead to the persistent inflammation seen in chronic wounds and diseased states. In the field of tissue regeneration, new therapeutics are continuously being developed with the intent to modulate immune processes to improve normal and impaired wound healing. Biomaterials used in these therapies have varied effects on local chemokine signaling. Understanding these mechanisms will help us refine the use of biomaterials in wound healing therapeutics. This review will discuss the available literature on the effects of some of the most common natural and synthetic biomaterials used in cutaneous wound healing therapies on chemokine signaling.

Soft tissue wound healing occurs in three stages: inflammation, proliferation and maturation (Rittié, 2016). The inflammation stage starts from the onset of injury and lasts about seventy-two hours. During this stage, immune cells are attracted to the injured tissue to clear foreign material and microorganisms. In the proliferation stage, the injured components of the wound bed are rebuilt through the processes of angiogenesis, extracellular matrix (ECM) synthesis, and epithelialization. This phase is thought to last from days four to twenty-one post-injury (Childs and Murthy, 2017). In the final stage of wound healing, maturation, fibroblasts conduct collagen remodeling and strengthen the regenerated tissue. This process can take up to sixty days after injury to reach maximum strength but the entire process can last up to a year. Different cell types are predominant during each stage of wound healing, and their recruitment is largely dependent on cytokine and chemokine signaling. A brief overview regarding these signaling pathways in normal wound healing in humans will be discussed.

The inflammation stage starts with the formation of a platelet plug at the site of injury, which activates the coagulation cascade and recruits and amplifies cells to the injured tissue. Innate immune cells are predominant among cells recruited, and include neutrophils, monocytes, and mast cells. Exposed collagen at the injury recruits and aggregates platelets, which release platelet-derived growth factor (PDGF) and transforming growth factor (TGF) beta, two strongly chemotactic agents for neutrophils (Childs and Murthy, 2017). Neutrophils are the predominant cell type recruited initially during the inflammation stage. In addition to clearing microorganisms in the wound, neutrophils release inflammatory cytokines that lead to catecholamine release and vasoconstriction, enabling hemostasis. Neutrophils maintain their presence through a positive feedback loop through the secretion of the chemokine CXCL8 in large quantities, which in humans is considered the most potent neutrophil chemoattractant (Satish, 2015). CXCL1, 5, 6, and 7 are also chemotactic to neutrophils, although to a lesser extent, and are produced downstream by macrophages (Nakkala et al., 2021).

Following hemostasis, recruited mast cells release histamine, leading to vasodilation and increased blood flow to the injury site, which carries along additional immune cells. Mast cells secrete CXCL8 but secrete larger quantities of CCL2 and CCL4, along with smaller quantities produced by neutrophils, to recruit peripheral monocytes from the blood, bone marrow and spleen (Nakkala et al., 2021; Saleh et al., 2022). CCL2 is also produced by basal keratinocytes at the wound edge (Satish, 2015). Peripheral monocytes express the chemokine receptor CCR2, which binds CCL2, 7, 8, and 12, and differentiates them from tissue-resident monocytes which are recruited locally by chemokines CCL17, 18, and 22 (Saleh et al., 2022). Both types of monocytes differentiate into macrophages and clear dead cells and necrotic tissue from the injury site.

Peripheral monocytes are differentiated into macrophages in what is considered the “classical activation” pathway (Satish, 2015; Sokol and Luster, 2015). Tissue-resident monocytes are differentiated into macrophages by local innate immune cells that recognize the damage-associated molecular patterns (DAMPs) released by dead or dying cells, in what is considered the “alternative activation” pathway. Classically activated macrophages were previously categorized as “M1” pro-inflammatory phenotypes while alternatively activated macrophages were categorized as “M2” anti-inflammatory phenotypes (Li and Bratlie, 2021). More recently, the literature suggests that both peripheral and resident macrophages are dynamic in M1 and M2 phenotypes and may also exist somewhere in between this dichotomy. Therefore, we will focus on how the dominant macrophage phenotype, rather than the origin of the monocyte, affects wound healing.

In normal wound healing, the predominant macrophage phenotype transitions from M1 to M2 near the end of the acute inflammatory phase, around seventy-two hours following injury (Martin and García, 2021). This marks the beginning of the proliferative phase, during which cellular recruitment and signaling are largely focused on angiogenesis, ECM synthesis, and epithelialization. While macrophages are the predominant cell-type in this stage of wound healing, other cell types synergistically support these processes.

Endothelial cells release vascular endothelial growth factor (VEGF) and chemokines CCL2 and 5 and CXCL1, 8, 9, and 10 to promote angiogenesis and leukocyte migration (Satish, 2015; Childs and Murthy, 2017). While the role of CCL2 in neutrophil chemotaxis was previously discussed, in the proliferative phase, CCL2 also promotes angiogenesis, epithelialization and collagen formation, as demonstrated by a deficiency in these processes in CCL2 knock-out mice (Satish, 2015). Aside from endothelial cells, mesenchymal stromal cells (MSC) play a major role in this stage of wound healing. The chemokine CXCL12 released from MSC has been widely studied on its effect on stem cell migration and homing to the injury site (Satish, 2015). Additional processes include continued PDGF release from platelet degranulation which leads to promotion of collagen formation from fibroblasts. Fibroblasts also release growth factors that stimulate keratinocytes to increase epithelialization. Maximum collagen deposition occurs in the proliferative phase at day twenty-one (Childs and Murthy, 2017).

**FIGURE 1:**
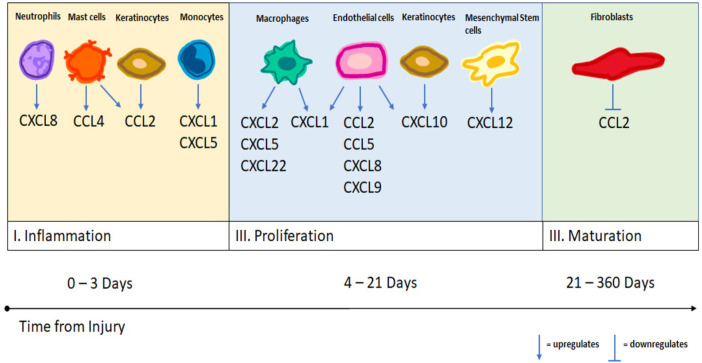
A summary of different cell types and their upregulation and downregulation of specific chemokines as demonstrated by the literature.

In the final stage of wound healing, the maturation stage, fibroblasts convert the initially deposited collagen III into collagen I. This remodeling of collagen helps strengthen the regenerated tissue and usually maximizes at forty-two to sixty days after injury. Fibroblasts are primarily recruited by PDGF, TGF-B, and VEGF (Nakkala et al., 2021). There is far less in known about the role of chemokine signaling in this stage of wound healing. The majority of the literature focuses on the interaction between fibroblasts and the chemokines CCL2 and CCL3. Fibroblasts cleave previously released CCL2, which generates antagonists to CCR2 and inhibits further leukocyte chemotaxis (Satish, 2015). This stage may be critical as previous studies have shown that co-cultures of fibroblasts and macrophages produce much larger quantities of CCL2 and CCL3 than isolated fibroblasts (Zickus et al., 1998; Zeng and Chen, 2010). Continued cell-cell interactions between macrophages and fibroblasts may lead to the formation of hypertrophied scar, keloid and fibrosis (Childs and Murthy, 2017). See Figure 1 for a summary of the chemokine modulation by different immune cell types at each stage of wound healing.

Theoretically, disruptions to any of these stages of wound healing could contribute to delayed wound healing. Persistence of the inflammation stage, however, is thought to contribute the most to delayed wound healing in diabetic populations (Seibold et al., 1985; Rafehi et al., 2011; Moura et al., 2014). For example, in normal wound healing, the M1 response allows for hemostasis and inflammatory cell recruitment and sets the foundation for the M2 response to sustain angiogenesis, stimulate cell growth and proliferation and ECM remodeling in the wound bed. While both phenotypes are necessary for appropriate wound healing, the timing of the transition is paramount to ensure progression of healing. In diabetic mice, for example, the M1 phenotype has been shown to persist beyond three days after wounding, instead of transitioning to M2 as in normal wounds (Louiselle et al., 2021).

Inappropriate regulation of chemokines in diabetic populations could contribute to this delay in progression. RNA sequencing has shown that diabetic skin upregulates CCL2, CCL7, CCL9, CCL12, CCL20, CXCL2, and CXCL15 as compared to normal healthy skin (Kuang et al., 2021). Among these, CCL2 has additionally been shown to be increased in diabetic wounds (Kuang et al., 2021). The role of CCL2 in maintaining immune cell chemotaxis in all three stages of wound healing has been previously discussed. Diabetic mice and their wounds have also been shown to be deficient in CXCL12 and show improved wound healing when treated exogenously with this chemokine (Badillo et al., 2007; Restivo et al., 2010). Stem cell homing is a crucial part of the proliferative stage in wound healing.

Given the dysregulation of chemokine signaling in the transition from the inflammatory to the proliferative stage, this has become an area of interest in the design of biomaterials for therapeutic use in wound healing. Since this transition correlates with the M1 to M2 phenotype switch in macrophages, some researchers have been steered toward seeking biomaterials that promote the expression of M2, the more “desirable” macrophage phenotype in wound healing. However, it should be noted that M2 macrophages may also be responsible for macrophage fusion, fibrosis, and the foreign body response (FBR), and their excessive upregulation could lead to unwanted scar formation (Martin and García, 2021). Additionally, as discussed before, some degree of inflammation is required to ensure successful wound closure.

One way of examining whether a biomaterial induces an M2 phenotype is to modulate its effect on chemokine signaling. Unfortunately, a lot of our knowledge about chemokine signaling in wounds is dependent on animal models, which is complicated by the fact that some chemokines expressed in humans do not exist in animal models and *vice versa*. And additionally, while the roles of some chemokines like CCL2, CCL5, CXCL8, and CXCL12 have been somewhat elucidated, the majority of chemokines are still not well understood in their role in wound healing, and studies vary in their categorization of them as pro- and/or anti-inflammatory. In general, CXCL4, 6, 7, 8, 9, 10, and 11, CCL1, 2, 3, 4, 5, 7, 11, 12, 20, 24, and 26, XCL1 and 2, and CX3CL1 are associated with pro-inflammatory effects, with downstream effects of neutrophil, eosinophil, basophil, and leukocyte recruitment. The chemokines CXCL12, 13, and 21 and CCL8, 13, 14, 17, 18, 19, and 27 are often associated with anti-inflammatory effects with downstream effects of stem cell homing, cell proliferation, angiogenesis and ECM remodeling (Li and Bratlie, 2021; Martin and García, 2021). While many of these chemokines share both pro-inflammatory and anti-inflammatory effects, the chemokines CXCL1, 2, and 5 and CCL22 appear to facilitate both more than others. A summary of chemokines and their known downstream effects on signaling and immune function in cutaneous wounds is displayed in Table 1 **.**

**TABLE 1:** A list of chemokines with presumed signaling profiles and immune functions in skin wound healing. Th, T helper; NK cell, natural killer cell; DC, dendritic cell; LN, lymph node.

Chemokine	Other names	Pro-inflammatory signaling	Anti-inflammatory signaling	Primary immune functions
CXCL1	Gro-a	Satish (2015), Sokol and Luster (2015), Ridiandries et al. (2018)	Satish (2015), Ridiandries et al. (2018), Nakkala et al. (2021)	Neutrophil trafficking (Sokol and Luster, 2015), angiogenesis (Ridiandries et al., 2018)
CXCL2	Gro-b, MIP-2	Sokol and Luster (2015), Ridiandries et al. (2018)	Ridiandries et al. (2018), Nakkala et al. (2021)	Neutrophil trafficking (Sokol and Luster, 2015), angiogenesis (Ridiandries et al., 2018)
CXCL3	Gro-y	Sokol and Luster (2015)		Neutrophil trafficking (Sokol and Luster, 2015)
CXCL4	PF	Satish (2015), Ridiandries et al. (2018)		Procoagulant and hemostasis (Sokol and Luster, 2015; Ridiandries et al., 2018)
CXCL5	LIX	Satish (2015), Sokol and Luster (2015), Ridiandries et al. (2018)	Satish (2015), Sokol and Luster (2015), Ridiandries et al. (2018)	Neutrophil trafficking (Sokol and Luster, 2015), angiogenesis (Ridiandries et al., 2018)
CXCL6	GCP-2	Satish (2015), Sokol and Luster (2015)	Ridiandries et al. (2018)	Neutrophil trafficking (Sokol and Luster, 2015), angiogenesis (Ridiandries et al., 2018)
CXCL7	Nap-2	Satish (2015), Sokol and Luster (2015), Ridiandries et al. (2018)	Ridiandries et al. (2018)	Neutrophil trafficking (Sokol and Luster, 2015), angiogenesis (Ridiandries et al., 2018)
CXCL8	IL-8	Satish (2015), Sokol and Luster (2015), Ridiandries et al. (2018), Martin and García (2021), Nakkala et al. (2021)	Satish (2015)	Neutrophil trafficking (Sokol and Luster, 2015), angiogenesis (Satish, 2015)
CXCL9	Mig-9	Satish (2015), Sokol and Luster (2015)		Th1, CD8^+^, NK cell trafficking (Satish, 2015; Sokol and Luster, 2015)
CXCL10	IP-10, CRG-2	Satish (2015), Sokol and Luster (2015), Bünemann et al. (2018)	Satish (2015), Ridiandries et al. (2018)	Th1, CD8^+^, NK cell trafficking (Satish, 2015; Sokol and Luster, 2015), epithelialization (Ridiandries et al., 2018)
CXCL11	1-TAC	Sokol and Luster (2015)	Ridiandries et al. (2018)	Th1, CD8^+^, NK cell trafficking (Satish, 2015; Sokol and Luster, 2015), epithelialization (Ridiandries et al., 2018)
CXCL12	SDF-1		Satish (2015), Sokol and Luster (2015), Ridiandries et al. (2018), Nakkala et al. (2021)	Stem cell homing (Satish, 2015; Sokol and Luster, 2015; Ridiandries et al., 2018; Nakkala et al., 2021)
CXCL13	BLC, BCA-1		Sokol and Luster (2015)	B-cell and T-follicular helper migration to lymph node (Sokol and Luster, 2015)
CXCL14	BRAK			DC skin homing (Sokol and Luster, 2015)
CXCL21			Nakkala et al. (2021)	Angiogenesis (Nakkala et al., 2021)
CCL1	TCA-3	Satish (2015), Ridiandries et al. (2018)		Th2 and Treg trafficking (Satish, 2015; Ridiandries et al., 2018; Nakkala et al., 2021)
CCL2	MCP-1	Satish (2015), Sokol and Luster (2015), Bünemann et al. (2018), Ridiandries et al. (2018), Martin and García (2021), Nakkala et al. (2021)	Satish (2015), Ridiandries et al. (2018)	Monocyte trafficking (Sokol and Luster, 2015), ECM remodeling (Ridiandries et al., 2018)
CCL3	MIP-a/b	Bünemann et al. (2018), Ridiandries et al. (2018)	Ridiandries et al. (2018)	Macrophage and NK-cell trafficking (Sokol and Luster, 2015), ECM remodeling (Ridiandries et al., 2018)
CCL4	MIP-1	Satish (2015), Bünemann et al. (2018), Ridiandries et al. (2018), Nakkala et al. (2021)		Macrophage and NK-cell trafficking (Satish, 2015; Sokol and Luster, 2015; Bünemann et al., 2018; Ridiandries et al., 2018)
CCL5	RANTES	Satish (2015), Ridiandries et al. (2018)		Macrophage and NK-cell trafficking (Satish, 2015; Sokol and Luster, 2015; Ridiandries et al., 2018)
CCL7	MCP-3	Bünemann et al. (2018), Ridiandries et al. (2018)		Monocyte trafficking (Sokol and Luster, 2015; Bünemann et al., 2018; Ridiandries et al., 2018)
CCL8	MCP-2		Sokol and Luster (2015)	Th2 response (Sokol and Luster, 2015)
CCL11	Eotaxin	Satish (2015), Bünemann et al. (2018)		Eosinophil and basophil trafficking (Sokol and Luster, 2015)
CCL12	MCP-5	Sokol and Luster (2015)		Monocyte trafficking (Sokol and Luster, 2015)
CCL13	MCP-4		Sokol and Luster (2015)	Th2 responses (Sokol and Luster, 2015)
CCL17	TARC		Sokol and Luster (2015), Bünemann et al. (2018)	Th2 response and cell migration, skin homing (Sokol and Luster, 2015; Bünemann et al., 2018)
CCL18	PARC, DC-CK1		Sokol and Luster (2015)	Th2 response, skin homing (Sokol and Luster, 2015)
CCL19	MIP-3b		Sokol and Luster (2015)	T-cell and DC homing to LN (Sokol and Luster, 2015)
CCL20	MIP-3a	Bünemann et al. (2018)		Th17 responses (Sokol and Luster, 2015)
CCL21	SLC, 6CKine		Sokol and Luster (2015), Ridiandries et al. (2018)	T-cell and DC homing to LN (Sokol and Luster, 2015)
CCL22	MDC	Satish (2015)	Sokol and Luster (2015)	Th2 response and cell trafficking (Sokol and Luster, 2015), lymphocyte recruitment (Satish, 2015)
CCL24	Eotaxin-2, MPIF-2	Sokol and Luster (2015)		Eosinophil and basophil trafficking (Sokol and Luster, 2015)
CCL25	TECK			T-cell homing to gut (Sokol and Luster, 2015)
CCL26	Eotaxin-3	Sokol and Luster (2015)		Eosinophil and basophil trafficking (Sokol and Luster, 2015)
CCL27	CTACK		Bünemann et al. (2018), Ridiandries et al. (2018)	Stem cell homing (Ridiandries et al., 2018), epithelialization (Bünemann et al., 2018)
XCL1	Lymphotactin, SCM-1a	Bünemann et al. (2018)		Cross presentation by CD8^+^ (Sokol and Luster, 2015)
XCL2	SCM-1B	Sokol and Luster (2015)		Cross presentation by CD8^+^ (Sokol and Luster, 2015)
CX3CL1	Fracktaline	Sokol and Luster (2015)		NK, monocyte, T-cell trafficking (Sokol and Luster, 2015)

Immunomodulatory biomaterials can manipulate the body’s immune response in a favorable way by promoting tissue regeneration. Studying the effects biomaterials have on chemokine and cell type expression can provide insight into the mechanism of their therapeutic effect. There are multiple physical and chemical properties that have been identified as promoting an M2 phenotype for example, such as soft surface topography, 50 nm porous structures, aligned nanofibers, and hydrophobic surfaces (Shen et al., 2021). Augmenting the anti-inflammatory response to the benefit of a local wound environment may be as important as preventing the FBR, which has been also attributed to the M2 phenotype (Martin and García, 2021; Villarreal-Leal et al., 2021). Biomaterials with a self-limiting immunomodulatory effect may be the most desirable, and those that can imitate the ECM have been shown to have more success in this regard (Villarreal-Leal et al., 2021). In this review, we will discuss commonly used and researched natural and synthetic biomaterials used in wound healing and how they may be modulating chemokine signaling.

## Natural biomaterials

Two main groups of biomaterials will be discussed in this section: polysaccharides and silk fibroin polymers. Polysaccharides are natural macromolecule polymers composed of monosaccharides bound by glycosidic linkages and can be derived from plants, like alginate, and from animals, like chitosan and glycosaminoglycan (GAG) (Li and Bratlie, 2021). These polymers mimic the structure and microenvironment of ECM, which has made them a popular choice of biomaterial in tissue engineering. Still, biomaterials made from polysaccharides cannot escape detection from the body’s immune system. Macrophages can identify polysaccharides through toll-like receptors (TLR) TLR4 and TLR2, which bind glycosyl ligands and activate tumor necrosis factor receptor-associated factor (TRAF) 6 or toll/interleukoin-1 receptor domain-containing adaptor protein inducing interferon beta (TRIF) pathways to eventual nuclear factor kappa-light-chain-enhancer of activated B (NFKB) activation. Macrophages with surface mannose receptors can also bind and phagocytose polysaccharide ligands (Li and Bratlie, 2021).

GAGs, chitosan and alginates are three polysaccharides that have been used in the construction of various therapeutics for wound healing. Each has different inherent physiochemical properties that can be modified to upregulate or downregulate certain chemokines. In this section, we will discuss the general effects of these three polysaccharides on chemokine signaling in relation to wound healing, as well as the effects of various modifications.

Silk fibroin is another natural biomaterial commonly used in wound healing. Fibroin has significant mechanical strength properties and its degradation products are considered non-toxic and potentially even beneficial in wound healing (Arkhipova et al., 2016). The same cross-linking of fibroin sheets that give it strength, however, can make it susceptible to enzymatic degradation (Lehmann et al., 2022). And when made into particles, there is some concern that fibroin may elicit an inflammatory response due to their smaller size (Cui et al., 2013). Both fibroin sheets and particles have been developed as therapeutics in wound healing and have been shown to alter chemokine expression. These effects will be discussed further in this section.

### Glycosaminoglycans

GAGs are naturally occurring negatively charged linear polysaccharides in the ECM of every mammal. Variations on the repeated core disaccharide unit and glycosidic linkages determine the major groups within GAGs - heparin sulfate, hyaluronic acid, chondroitin sulfate, dermatan sulfate and keratan sulfate (Sodhi and Panitch, 2021). Except for keratan sulfate, which is predominantly expressed in the cornea, bone and cartilage, all these groups have demonstrated potential uses in cutaneous wound healing (Plichta and Radek, 2012). GAGs are appreciated for their ability to support the ECM through cell hydration and structural scaffolding but may also play a critical role in cell signaling. Their physical and chemical properties can strongly influence chemokine signaling, and in turn, cell proliferation, differentiation, and migration.

Distribution of chemokines in tissue is largely dependent on their complexation with the GAGs of the ECM. Chemokines bind GAGs via both non-specific long-ranging electrostatic interactions and specific molecular binding between positive charges of amino acid residues on chemokines and negatively charged sulfate and carboxylate moieties on the GAG chain (Lohmann et al., 2017; Nordsieck et al., 2018; Schirmer et al., 2021). When sulfated GAGs are arranged into a 3D structure such as a hydrogel, the cumulative negative charges of sulfate moieties create an overall negative global charge that attracts positively charged proteins like chemokines; this is known as the integral charge density, and is also dependent on the concentration of GAG (Schirmer et al., 2021). The local charge density of a single GAG chain within the hydrogel, which is dependent on the number of sulfate moieties in that chain, is another factor that determines which chemokine will bind. The type of glycosidic linkage in a GAG can also modify the electrostatic interactions with chemokines (Nordsieck et al., 2018). By modifying the integral and local charge densities of a GAG hydrogel, along with their glycosidic linkages, researchers have sought to design therapeutics that attract specific chemokines (Schirmer et al., 2021; Lehmann et al., 2022).

The ability of GAG to interact and bind with CXCL8 has been specifically investigated at some length. Truncation studies of CXCL8 have shown that five positively-charge residues in the C-terminal alpha helix play the most important role in binding with sulfated moieties in GAGs, although there are a few residues on the N-loop that also contribute. Using this knowledge, the binding affinities of these domains in CXCL8 with GAGs of different charge densities was studied. When compared to heparin, hyaluronic acid was shown to have far less binding affinity to CXCL8, which likely results from its lower charge density. The authors of this study conclude from these results that hyaluronic acid may be a better choice among GAGs in wound healing therapeutics, since its moderate ability to sequester CXCL8 may also translate to reduced sequestering of potentially beneficial chemokines (Nordsieck et al., 2018).

The use of charge density to bind CXCL8 was further investigated by Lohmann et al. in the creation of a biohybrid hydrogel made from star-shaped polyethylene glycol (starPEG) crosslinked with heparin (Lohmann et al., 2017). Heparin sulfate has the highest anionic charge density among all GAGs and the number of sulfated moieties on heparin correlates with its anionic charge density. In this study, starPEG-heparin hydrogels with various degrees of sulfate saturation were created maximizing integral and local charge densities that would attract the positive charge densities of CCL2 and CXCL8. *In vitro* evaluation of binding kinetics revealed that while the location of the sulfate affected binding of CCL2, the overall sulfate saturation had the biggest effect on CXCL8 binding. These findings translated to observations made when the hydrogel was placed in a medium conditioned to resemble chronic wounds; increased chemokine binding was observed in gels with higher heparin sulfate saturation.

When applied to a murine model, starPEG heparin gels were found to decrease levels of CCL2, CXCL8 and even CXCL1 in the wounds of mice at days 5 and 10 after injury (Lohmann et al., 2017). Comparable results were found when this experiment was repeated on diabetic mice and on diabetic mice with *Staphylococcus aureus* infected wounds. CCL2, CXCL8 and CXCL1 are all in greatest abundance during the inflammatory phase and their reduction at days 5 and 10 demonstrates the potential mitigation of what would otherwise be persistent activation of these chemokines, which would prevent transition to the proliferative stage. From these studies, the authors developed a wound contact layer using a hybrid percent of starPEG-heparin that maximizes binding capacity of proinflammatory chemokines while minimizing unwanted sequestering of growth factors. They demonstrated improved wound healing rates compared to controls in porcine wound models at day fourteen and beyond (Schirmer et al., 2021). Comparable results have been seen in biomaterials made from highly sulfonated hyaluronic acid, with reductions in CXCL1 and CCL2 in a murine wound healing model (Hauck et al., 2021).

The starPEG-heparin hydrogel has also been used to integrate a desired chemokine, like CXCL12, for therapeutic delivery, by modifying the amino acids in the heparin-binding regions of CXCL12 (Spiller et al., 2019). CXCL12 is a chemokine known for its potent stem cell homing abilities, and application of both this chemokine and mimetics have been shown previously to stimulate wound healing (Badillo et al., 2007; Xu et al., 2022a). Another group integrated CXCL12 into their GAG therapeutic by using a hybrid polyethyleneimine (PEI)-CXCL12 containing collagen-chondroitin sulfate scaffold (Laiva et al., 2021).

These studies highlight a few ways in which understanding interactions between GAGs and chemokines may allow us to manipulate the properties of GAG-based biomaterials to maximize their therapeutic potential in wound healing. Variations on both local and integral charge densities of GAGs appear to have the biggest influence on binding mechanics with chemokines. By manipulating these properties, this biomaterial has been used to both sequester proinflammatory chemokines and also administer anti-inflammatory chemokines. While there is ongoing research on the use of natural GAGs in wound healing biomaterials, there is perhaps even a greater abundance of investigations on naturally occurring GAG-mimetics. These include biomaterials like chitosan and alginate, which have been used extensively in the development of wound healing therapies. Like the naturally occurring heparin, the physical and chemical properties of these biomaterials have also been found to affect their impact on chemokine signaling and will be discussed in the next section.

### Chitosans

Chitosan is a deacetylated derivative of the naturally occurring polymer, chitin, which is found in the exoskeletons of crustacea, the cell wall of fungi, insect cuticles and in algae (Aranaz et al., 2021). Like GAG, chitosan is a linear polysaccharide, but instead carries a positive charge. The charge density of chitosan is dependent on the degree of acetylation and the pH of media, while its solubility is dependent on the degree of acetylation and molecular weight. In general, chitosan biomaterials are more soluble in acidic aqueous solutions and have greater solubility with lower molecular weights (Aranaz et al., 2021). There have been multiple studies demonstrating the ability of chitosan to stimulate wound healing (Aranaz et al., 2021). However, their relatively poor solubility in aqueous solutions at neutral biological pH has presented challenges for use in biomedical applications. Additionally, there is evidence that chitosan can be proinflammatory and cytotoxic in certain conditions.

While the mechanism by which chitosan supports wound-healing is still not well understood, some hypothesize that chitosan, and in particular, low molecular-weight (LMW) chitosan, is able to stimulate the M2 phenotype. Guzman-Morales et al. test this hypothesis by applying a LMW chitosan at 40 kDa to mouse bone marrow-derived macrophages (BMDM) *in vitro* and assessing expression of arginase-1, an essential mediator in the production of polyamine in prolines of murine M2 phenotypes (Guzmán-Morales et al., 2011). Levels of arginase-1 production in BMDM treated with chitosan were elevated as compared to those treated with latex beads or untreated BMDM, but not significant when compared to those treated with IL-4, which was meant to stimulate a positive control for M2 activation. When examining chemokine expression, they showed that LMW chitosan induced mild increases in the production of CCL2, 3, and 5, which are all considered more pro-inflammatory chemokines. These increases were insignificant, however, when compared to BMDMs stimulated with lipopolysaccharides (LPS), a known inflammatory molecule (Guzmán-Morales et al., 2011). They determine that LMW chitosan does not incur a significant proinflammatory chemokine reaction, making it an effective biomaterial in therapeutic development. It must be considered, however, that this study was done *ex vivo*, which limits its interpretation for clinical use.

Modifications to the chemical properties of chitosan may affect chemokine signaling. Transwell migration assays performed on chitosan with varying extents of acetylation were conducted using a differentiated model cell line (HL60-PMN) to assess the effect of chitosan charge and hydrophobicity on neutrophil migration. They found that neutrophil migration increased with increasing chitosan-N-acetylation (Park et al., 2009). Additionally, increasing levels of CXCL8 were detected when HL60-PMN cells were exposed to chitosan of increasing N-acetylation. The N-acetylation of chitosan reduces its positive charge and also makes it more hydrophobic. Given the stronger correlation between N-acetylation and hydrophobicity, the authors propose that it is the hydrophobic nature of the chitosan that is influencing its ability to promote CXCL8 secretion. The mechanism behind this is not well understood, although the authors postulate that since neutrophils have affinity for hydrophobic surfaces, the chitosan gel may be prolonging the interaction between neutrophils and injured cells. Given that persistent CXCL8 secretion has been shown to exist in chronic wound environments, the authors suggest that biomaterials using chitosan with reduced N-acetylation would be preferrable in the development of wound healing therapeutics.

Modifying chitosan with other compounds can also affect chemokine signaling. A chitosan-dextran based gel was developed for use as a post-surgical aid in endoscopic sinus surgery. This gel uses a version of chitosan with succinyl added to the N-glucosamine units of chitosan, which allows the chitosan to be soluble in aqueous solutions at physiological pH. Dextran serves as a cross-linker to the abundant amino acid groups in succinyl-chitosan. In cytotoxicity studies, dextran was found to be the bioactive component of this gel, with cytotoxic effects on epithelial cells and macrophages seen in culture. When stimulated with this chitosan-dextran gel, dermal fibroblasts were shown to produce significantly less CXCL8 than controls. This effect was noted for the succinyl-chitosan without dextran as well, although with less prominent effect. In this study, the local toxicity of the chitosan-dextran gel is presumed to target unwanted fibroblasts and in turn reduce inflammatory chemokine secretion and subsequent undesired FBR and scar formation (Aziz et al., 2015). One caveat is that in this study the production of CXCL8 was only examined for dermal fibroblasts, which is not the immune cell type most associated with this chemokine’s excess production and while similar results were seen in a reduction of TNF-a production by macrophages, it would be interesting to know whether the gel similarly affects macrophages CXCL8 secretion.

Carboxymethylation is another chemical modification to chitosan with potential significance on chemokine signaling. In one study, authors describe the use of a chitosan gel modified by N-carboxymethylation to obtain higher solubility, higher viscosity and lower toxicity on second degree burn wounds on rats. In this study, wounded rats received daily topical treatment to their wound for thirty days with either saline or N-carboxymethylated chitosan gel. At day sixteen, the treated group’s wound healing rate was significantly improved over controls, with grossly less contract and scar (Chang et al., 2013). On histology, significant fewer polymorphonuclear leukocytes (PMN) were detected in the treated group and on quantification of cytokines in wound tissue by ELISA, levels of TGF-B and TGF-a were increased and decreased, respectively. Interestingly, when examining CXCL8 expression, there was overall no difference between the treated and control group. In the treated group, CXCL8 levels peaked at a higher level than controls between days three and five. Unlike the last example, in this study, the carboxymethylated chitosan is shown to increase fibroblast proliferation, which the authors use to explain these results.

The authors argue that augmenting CXCL8 levels can contribute to accelerated wound healing through promotion of angiogenesis (Chang et al., 2013). The ability of CXCL8 to promote pro-inflammatory *versus* angiogenic effects in wound healing is likely dependent on the timing of its release. Although it is detected in the proliferation stage, this chemokine predominates during the inflammatory phase (Ridiandries et al., 2018). In chronic wounds, the persistence of CXCL8 is thought to occur pathologically. In wounds treated with carboxymethylated chitosan, although there is augmented CXCL8 in between days three and five, levels are seen normalizing thereafter, which correlates with an appropriate transition from inflammatory to proliferative stage (Chang et al., 2013). A transient increase in CXCL8 at the appropriate stage may benefit wound healing.

Chitosan can also be used a therapeutic biomaterial by embedding it with bioactive compounds, such as stem cells, which have been extensively documented in their ability to benefit wound healing. A study investigating the effects of self-assembled adipose stem cells (ASCs) spheroids on the healing of full thickness rat skin wounds used chitosan-hyaluronan membranes for ASC self-assembly. After seventy-two hours, the wound area was significantly smaller in rats treated with the ASC-containing chitosan-hyaluronan gel. On evaluation of gene expression, these wound tissues expressed more VEGF, CXCR4, matrix metallopeptidase 1 (MMP1) and CCL2 than those treated with hydrogels containing just single cells grown on tissue-culture polystyrene (Hsu and Hsieh, 2015). VEGF and CXR4 are known mediators of angiogenesis and MMP1 is essential in ECM turnover (Satish, 2015). While CCL2 has been identified as predominantly a pro-inflammatory chemokine (Kuang et al., 2021), it has been shown to have anti-inflammatory and proangiogenic effects as well (Satish, 2015; Ridiandries et al., 2018). Its increase at seventy-two hours could benefit the proliferative stage of wound healing. One challenge in interpreting the results of this study is distinguishing which of the two variables—the material in which ASCs are seeded and the ASC geometry—are responsible for the demonstrated changes.

CXCL12 has also been embedded into chitosan. In one study, a hybrid hydrogel made from chitosan and polyvinyl acetate (PVA) that is chemically crosslinked by glutaraldehyde and loaded with CXCL12 is shown to steadily release CXCL12 from hour three to forty-eight hours. When applied every 2 days for a total of four applications on non-diabetic rats with full thickness square wounds, the gel showed a trend to accelerate wound closure, with significance at days seven and eleven (Xu et al., 2022b). On gross examination, treated wounds did not have the standard scar associated with the controls. Another group created a similar chitosan-based biomaterial, except using genipin, a naturally occurring antibacterial and anti-inflammatory compound found in Gardenia jasminoides, to crosslink chitosan amine groups in its scaffold. These chitosan-genipin scaffolds incorporated with CXCL12 were applied in an excisional wound model created in healthy and diabetic rats. Compared to wounds treated with chitosan-genipin alone, the commercial wound dressing Comfee, and gauze, the wounds treated with the chitosan-genipin-CXCL12 scaffold showed improved wound healing rates at days four and seven. In diabetic rats, wounds treated with chitosan-genipin-CXCL12 scaffold had 58.8% increase in recovery rate as compared to controls at day seven (Yao et al., 2020).

Overall, the chitosan alone does not appear to have a significant inflammatory effect on chemokine signaling. The use of low-molecular weight chitosan and modification with reduced N-acetylation groups, increased N-carboxymethylation groups and conjugation with dextran all have been shown to have some immunomodulatory benefit in wound healing. Two of the studies discussed suggest that chitosan is altering chemokine expression from fibroblasts, which may provide a mechanistic explanation behind its benefits, however further studies on multiple cell types at various stages of healing are needed to better characterize the effect these modifications have on the immune system.

### Alginates

Alginate is another polysaccharide of (Satish, 2015; Sokol and Luster, 2015; Rittié, 2016; Childs and Murthy, 2017) linked alpha-D-mannuronic acid (M) B-L-guluronic acid (G) and is derived from marine and bacterial sources. The ratio and distribution of M and G residues in addition to the molecular weight of the alginate compound determines its physiochemical properties. Regions rich in G residues enable cross-linking with cations in the formation of alginate hydrogels (Stenvik et al., 2012). Alginate hydrogels and dressings have long been used in wound care given their ability to absorb wound exudate, retain wound moisture, and lower bio-burden, in addition to their hemostatic properties and good permeability for gas and fluid exchange (Fischer et al., 2017). The vast majority of therapeutic biomaterials developed from alginates use marine alginates derived from seaweed.

The effects of alginate on the inflammatory response were studied using a biodegradable hydrogel made from oxidized alginate and marine gelatin. Primary macrophages and fibroblasts from both diabetic and wild-type mice were cultured with the hydrogel and analyzed using microarray for cytokines and chemokines. In wild-type mice, the chemokines upregulated with exposure to the alginate gel were CXCL4, CCL3, and CCL12, and those downregulated were CXCL5, CCL1, 2, 5, and 11 (Zeng and Chen, 2010). CXCL4 is considered a procoagulant, and essential for the hemostatic portion of the inflammatory stage. CCL3 can play a role in ECM remodeling (Ridiandries et al., 2018) and CCL1, CCL5 and CCL11, which are downregulated, are all generally pro-inflammatory chemokines (Satish, 2015; Bünemann et al., 2018; Ridiandries et al., 2018). The upregulation of chemokines associated with the proliferative phase and the downregulation of chemokines associated with the inflammatory phase suggest a mechanism by which alginate gel may accelerate wound healing (Zeng and Chen, 2010).

Another study suggest that alginates improve wound healing by upregulation of CXCR7-CXCL12 axis. A microarray analysis showed that calcium-free alginate is associated with upregulation of CXCR7 and its ligand, CXCL12, in keratinocytes. Other chemokine receptors for CXCL12, CXCR3 and CXCR4, and the other ligand for CXCR7, CXCL11, were not upregulated. This suggests that alginates work exclusively on the CXCR7-CXCL12 axis alone, although the exact mechanism is unknown. This effect was shown to be independent of calcium content. Calcium cations are commonly used to crosslink alginates into a hydrogel and have been shown to induce differentiation and inhibit proliferation in keratinocytes. These effects are mitigated with the use of low-calcium alginates, and non-existent with the use of calcium-free alginates. This data suggests that among alginate derivatives, low-calcium and calcium-free alginate derived products may be more suitable for use in wound healing (Stenvik et al., 2012). And similar to GAGs and chitosan, alginate hydrogels have also been used to deliver exogenous CXCL12. A study conducted on full thickness swine wounds showed the use of an alginate hydrogel patch containing CXCL12 led to faster healing rates than controls by day nine, with little evidence of scarring (Rabbany et al., 2010).

In a separate study, wound pads made from both marine and bacterial alginates were tested for their binding capacity for pro-inflammatory cytokines and chemokines, including CXCL8. *In vitro* studies showed all alginate-based wound pads led to a 90%–96.7% reduction in CXCL-8. The mechanism by which alginate sequesters CXCL8 is unknown, but the authors postulate that the anionic charge in polymer matrices of alginate may attract positively charged chemokines through non-covalent interactions (Fischer et al., 2017). This theory is similar to the hypothesized interactions between GAGs and chemokines.

Overall, alginates are a promising biomaterial for the development of therapeutics in wound healing given their demonstrated ability to upregulate anti-inflammatory chemokines like CXCL12 and downregulate pro-inflammatory chemokines like CXCL8. Still, more data is needed regarding the effects of alginate-based therapies *in vivo*.

### Silk fibroin

Silk fibroin is a non-toxic biocompatible natural polymer derived from silk from the silkworm *Bombyx mori*. There are several studies showing the use of silk fibroin-derived materials in wound healing. Our group developed a highly viscous nanosilk fibroin solution that was shown to increase tensile strength when applied on human skin and reduce levels of IL-6 in murine diabetic wounds (Niemiec et al., 2020). Like other polymers, fibroin and its derivatives can vary in their effects on inflammation and chemokine signaling depending on their physiochemical properties.

While fibroin scaffolds are generally considered bioinert, small fibroin degradation products may induce a mild inflammatory response. This is demonstrated by one study showing that murine wounds treated with microcarriers containing fibroin matrices had prolonged high-level expression of inflammatory cytokines at day ten as compared to controls treated with phosphate buffer saline (PBS). On evaluation of wound healing, however, both groups treated with fibroin-containing microcarriers had decreased wound contraction, reduced scar formation, and restoration of adipose tissue, increased muscle tissue and increased blood vessel and nerve outgrowth (Arkhipova et al., 2016). The expression of proinflammatory cytokines past the expected 5 days of the acute inflammatory phase in the context of accelerated wound healing draws into question whether this is an effect, cause, or merely an association with fibroin structure and size.

The addition of gelatin to these fibroin microparticles further improved wound healing rates and tissue regeneration when compared to those without it. Similarly as before, tissue samples treated with gelatin-fibroin particles showed increased expression of proinflammatory cytokines and chemokines, with particular increases in CXCL1 and CXCL2 on day 10 (Arkhipova et al., 2016). The persistence of elevated levels of pro-inflammatory chemokines and cytokines has usually been associated with chronic wounds; however these authors conclude that controlled augmentation of inflammation in acute stages of wound healing could potentially improve healing. Additionally, there are proangiogenic effects associated with CXCL1 and CXCL2, and their increased release at day 10 may be crucial to facilitating the proliferative stage of wound healing (Ridiandries et al., 2018).

The previous study discusses the use of microspheres, or spherical scaffolds, as a mean of fibroin delivery. Another group investigates the physiochemical properties of cross-linked *versus* “non-woven” fibroin-base scaffolds. One advantage to a “non-woven” fibroin scaffold is it is less stiff, which can expand its applicability in bioengineering. In this study, a non-woven fibroin scaffold is created from fibroin fibers 50 +/− 7 mm in length that are carded and hydroentangled, in which microfibers are twisted together by mechanical action of water. There is no formal cross-linking but electrostatic interactions among repetitive glycine-alanine and glycine-glycine sequences form weak bonds to shape the scaffold into a stable beta-sheet conformation. When used to seed adult human dermal fibroblasts (HDF), these fibroin-scaffolds enhanced secretion of CXCL1, 2, 3, and 8, and reduced secretion of CCL2 as compared to adult HDF seeded on polystyrene, a synthetic polymer (Hu et al., 2021).

The effects of chemokines vary depending on their interaction with one another and their timing of release in the wound healing process. Cells from this study were extracted from the scaffolds at hour seventy-two, which would correlate with the end of the inflammatory phase. The upregulated chemokines CXCL1, 2, 3 and 8 all have some association with pro-inflammatory signaling, however, CXCL1 and 2 also have proangiogenic effects and their upregulation may be critical to transitioning to proliferation (Ridiandries et al., 2018). CCL2 is generally identified as a pro-inflammatory chemokine and its downregulation by fibroin likely reduces monocyte recruitment, also advancing the transition from the inflammation to proliferative stage (Sokol and Luster, 2015; Ridiandries et al., 2018). One limitation of this study is that only dermal fibroblasts were seeded to these scaffolds, which means we are only getting one perspective on how one cell type would react to this biomaterial.

Fibroin is a protein polymer, as opposed to the polysaccharide polymers GAG, chitosan and alginate. The integration of fibroin into scaffolds and hydrogels has been shown to have varying effects in chemokine signaling. In gelatin, fibroin was shown to increase production of proangiogenic chemokines CXCL1 and CXCL2 towards the middle of the proliferative stage. In scaffolds, fibroin similarly induces and increases angiogenesis via upregulation of CXCL1 and CXCL2, while limiting further monocyte recruitment by downregulating CXCL8. There are limitations to what we can gain in our understanding of biomaterial and chemokine interactions from *in vitro* studies and further studies will need to be conducted regarding fibroin *in vivo*.

## Synthetic biomaterials

While natural biomaterials are praised for their biocompatibility, synthetic biomaterials are more easily modifiable, making them a desirable alternative in the development of wound-healing therapeutics. Like their counterparts, certain physiochemical properties of synthetic biomaterials have advantages in wound healing. The immunomodulation of chemokine signaling could be one explanation for this, and there has been research directed toward understanding these effects. For example, synthetic materials with hydrophobic surfaces have shown to reduce expression of inflammatory chemokines CXCL8 and CCL4, while among hydrophilic biomaterials, the same is true for those with cationic surfaces (Chang et al., 2008).

There are multiple synthetic biomaterials that are commonly used in wound-healing, including polyethylene glycol (PEG), poly(lactic-co-glycolic acid) (PLGA), and poly(methacrylic acid) (MAA). PEG is a hydrophilic ether-based polymer that has been used to make dressings designed for diabetic wounds (Mir et al., 2018). PLGA is a copolymer lactic acid and glycolic acid with excellent biocompatibility and biodegradability that has been used in multiple FDA-approved therapeutic devices. MAA is another petrochemical-based synthetic biomaterial that has been used in the industrial field for production of adhesives, paints, varnishes. More recent research has directed the use of MAA in the wound healing field due to claims of pro-angiogenic properties.

### Polyethylene glycol

Among synthetic polymers, PEG hydrogels have become increasingly used in biomedical applications in tissue regeneration given its biocompatibility and the versatility of its chemistry (Lin and Anseth, 2009). Most PEG-hydrogels are created via cross-linking and variations in cross-linking density as well as the incorporation of degradable linkers can modify the properties of these hydrogels. For example, loosely cross-linked PEG-hydrogels have superior water-containing capacity than “stiffer” gels, and the addition of poly(lactic acid) (PLA) can modify the degradation rate of PEG-hydrogels to allow for more sustained release profiles. Additionally, PEG-hydrogels have anti-fouling properties that allow them to repel non-specific protein absorption and cell-adhesion, and hence evade the capsule forming inflammatory reaction by the body (Lin and Anseth, 2009).

The effects of a “stiff” PEG-hydrogel and its ability to elicit a foreign body response is examined in a study by Saleh et al. (2022). Typically, “stiffer” PEG-hydrogels have been reported to produce more significant inflammatory and foreign body responses. PEG-hydrogel discs implanted in subcutaneous pockets between the hypodermis and a layer of muscle in mice were explanted at days two, fourteen and twenty-eight to evaluate acute inflammation, chronic inflammation, and fibrosis, respectively. Tissues were harvested and isolated using flow cytometry for Ly6chi macrophages by gating Ly6 and CX3CR1 positive cells and identified as peripheral monocytes recruited from the circulation. Ly6clo macrophages were gated with Ly6 negative and CX3CR1 positive cells and identified as tissue resident macrophages recruited to the site of injury. Tissue from uninjured mice was harvested for control Ly6clo macrophages and from their spleens for Ly6chi macrophages. The gene expression profiles of these macrophages were analyzed and compared.

At day two there was a significant increase in inflammatory gene transcription in both monocyte cell lines as compared to controls. Regarding chemokines, in tissue resident macrophages there was a significant increase in expression of CXCL1 and CXCL11. In circulating monocytes, CXCL1 and11 and CCL6, 7, and 24 were all expressed in significantly higher than in controls. Among those, only CXCL1 and CXCL11 have demonstrated anti-inflammatory profiles (Satish, 2015; Ridiandries et al., 2018), suggesting that the PEG hydrogel may actually invoke a more inflammatory response in both groups of monocytes. Interestingly, these increased levels of chemokines, in addition to other pro-inflammatory cytokines, remained upregulated in peripheral monocytes for almost twice as long as tissue resident macrophages. They repeated this experiment in CCR2−/− mice. CCR2 is expressed on circulating blood monocytes and leads to monocyte recruitment to injury sites via locally expressed CCL2. In this study, there was no significant difference in fibrous capsule formation between CCR2−/− mice and controls. Since tissue resident macrophages are still active in CCR2−/− mice, the authors conclude that tissue resident macrophages may play a greater role in capsule formation. They also conclude that the chemokine CCL2, which was not affected by the stiff PEG-hydrogel, is not the main chemokine responsible for fibrotic encapsulation (Saleh et al., 2022).

Photo-crosslinking is another method of cross-linking in PEG hydrogels that can modify their physiochemical profile. Kleinbeck et al. made an aqueous hydrogel from 30% PEG diacrylate and 20% gelatin that can be polymerized *in situ* under one minute of UV light application. They name this biomaterial a semi-interpenetrating network (sIPN) treatment. They apply the sIPN gel to porcine cutaneous partial-thickness wounds and compare response to treatment with Xeroform. They found that sIPN-treated wounds had relatively increased amounts of epithelialization on histology by 14 days and higher densities of dermal leukocytes and fibroblasts at twenty-one days. When comparing chemokines and cytokines there were no significant differences except for CXCL8, which was higher in the sIPN treated group at day seven. Given that this increase in CXCL8 occurs 7 days prior to the observed significant increase in epithelialization, the authors conclude that increased secretion of CXCL8 from higher dermal leukocyte and fibroblast densities during this critical time frame may be beneficial for epithelialization (Kleinbeck et al., 2010).

In another example of cross-linking variability, hyperbranched poly-L-lysine (HBPL) is used to crosslink hydrogel networks made from hydrophilic poly(PEGMA-co-GMA-co-Aam) (PPGA) polymers to explore its quorum sensing inhibition effects on infected diabetic wounds (Tu et al., 2022). HBPL has previously been shown to have defense against methicillin resistant *Staphylococcus aureus* (MRSA) by inhibiting quorum sensing and decreasing bacterial virulence and metabolic activity. The authors of this study synthesize PPGA polymers from PEG-methyl ether methacrylate (PEGMA), glycidyl methacrylate (GMA) and acrylamide (Aam). This hydrogel was cross-linked to HBPL-modified 2D MnO2 nanosheets via the glycidyl groups and pravastatin sodium loaded into each gel to obtain increased nitrous oxide synthetase activity. A study on diabetic mice with MRSA-infected wounds showed improved wound closure with 32.2% wound closure at day three compared to 8.5% in the controls. They also found decreased bacterial loads, reactive oxidative species (ROS) production, and myeloperoxidase activity.

IL-1B, IL-6, TNF-a, and CXCL1 had reduced expression in macrophages from wounds in hydrogel treatment groups at days three and six after treatment and IL-4 and IL-10, TNF-B and VEGF were increased, with the highest statistical significant differences at days six and fourteen (Tu et al., 2022). The reduction of CXCL1 using this biomaterial may be a part of the mechanism by which it reduces inflammation and accelerates wound healing. CXCL1 is a predominant neutrophil recruiter in the inflammatory stage of wound healing (Ridiandries et al., 2018). This PEG-based biomaterial had multiple physiochemical modifications to improve its wound healing potential. The HPBL both prevents bacterial proliferation through quorum sensing inhibition and stabilizes the colloidal formation of MnO2 nanosheets. The MnO2 nanosheets are able to protect against ROS and pravastatin increases synthesis of nitrous oxide. The authors suspect that decreased reactive oxidative species (ROS) and increased nitrous oxide prompts downregulation of pro-inflammatory chemokine CXCL1 in macrophages, although the potential mechanism behind this is not elaborated. This exemplifies the versatility of a biomaterial like PEG and how its modification can be optimized for its directed therapeutic use.

From the surveyed data, PEG can modulate immune responses in cutaneous wound through modifications of its “stiffness,” its cross-linking, and potential hybridization with other polymers. Among synthetic materials, PEG appears to have beneficial effects on reducing inflammatory and increase anti-inflammatory chemokine signaling, making it a popular choice in the design of wound-healing therapeutics.

### Poly(lactic-co-glycolic acid) or PLGA

PLGA is a polyester that has also been widely used in tissue regeneration due to its biodegradability, biocompatibility, and easily modified physiochemical properties. PLGA degrades under hydrolysis into lactic acid and glycolic acid monomers. There is evidence that the lactic acid byproduct can potentially even lead to angiogenesis and recruitment of EPCs. PLGA has been formulated into nanofibers, microspheres, hydrogels, and nanoparticles for use in wound healing. PLGA has good compatibility with other polymers, also making it a good vehicle for drug delivery (Chereddy et al., 2016).

Electrospinning is a technique used to produce micro and nano fibers from polymer solutions. In one study, PLGA fibers are created with electrospinning, coated with a poly(dopamine) (pDA) layer, and then loaded with recombinant PDGF-BB. Within the PDGF family, PDGF-BB is a potent mitogen for fibroblasts, keratinocytes, and vascular endothelium, and also stimulates the production of growth factors from macrophages. A recombinant form of PDGF-BB, known as Bercaplermin, is the only FDA-approved growth factor and has been used successfully for debridement and healing of diabetic ulcers. The addition, the pDA coating is thought to improve the hydrophilicity of PLGA fibers. When pDA/PLGA/PDGF-BB substrate is used on full thickness wounds in rats, wounds size and tissues measured at day seven were found to have 80% wound closure as compared to 44.71% of the controls, which subsisted of pDA/PLGA, PLGA/PDGF-BB and PLGA alone. This group also showed elevated expression of TGF-B and VEGF along with reduced expression of TNF-a (Yang et al., 2020). The success of Bercaplermin, however, has been limited by concurrent tumorigenic effects.

Another option for drug delivery using PLGA is the creation of microspheres, as exemplified by a study using these microspheres for therapeutic delivery of CXCL12 into wounds. The microencapsulation of CXCL12 into PLGA microspheres is done using a double-emulsion technique and the release profile of the CXCL12 is measured on a stimulated stem cell migration assay with porcine MSCs (Cross and Wang, 2011). This chemokine was similarly integrated into a PLGA scaffold by Thevenot et al. as an effort to recruit autologous stem cells to the PLGA implant site and potentially evade the fibrotic encapsulation that occurs in response to foreign bodies (Thevenot et al., 2010). At 2 weeks, CXCL12-incorporated PLGA scaffolds as compared to plain PLGA scaffolds showed upregulation of CXCL1, 2, 4, 9, 13, and 16 and CCL2, 3, 5, 9, 11, and 17. The chemokines CXCL5, 10, and 12 and CCL1, 12, 19, 20, 24, 25, and 27 were all downregulated. The most significant chemokines affected were the upregulation of CXCL2 and CCL4 and the downregulation of CCL1 (Thevenot et al., 2010). CXCL2 has proangiogenic effects and CCL4 and CCL1 are both potent macrophage recruiters (Ridiandries et al., 2018). These results demonstrate indirect “pro-inflammatory” “anti-inflammatory” effects of this biomaterial on chemokine signaling via the delivery of CXCL12.

Another group used PLGA microspheres to deliver both a peptide and ASCs. Velvet antler peptide (VAP) is a major bioactive component of velvet antler and an important Chinese traditional medicine ingredient that has been used for promotion of tissue repair and wound healing previously. The authors predicted that VAP would promote proliferation and migration of ASCs this was tested in full thickness murine wounds injected with hydrocortisone and stented open. Those mice treated with ASCs, VAP-PLGA, VAP-PLGA + ASCs had notably higher rates of wound healing than the control, with the highest rate in the VAP-PLGA + ASCs. VEGFR, CXCL12, CXCR4, TGF-B were all upregulated in treated groups, highest in VAP-PLGA + ASCs, and IL-1B, IL-18 and IL-6 were all downregulated, lowest in VAP-PLGA + ASCs group (Jiang et al., 2021). Activation of the CXCL12-CXCR4 axis may be the mechanism by which the synergy of the peptide and autologous stem cells in this biomaterial are promoting wound healing.

Variations on the structure of PLGA biomaterials as fibers or microspheres can influence the effect of PLGA on wound healing and chemokine signaling. More apparent in these studies, however, is the utility of PLGA as a means of delivery for therapeutic compounds into wounds. The therapeutic potential of PLGA itself and its effects on chemokine signaling warrants further studies.

### Poly(methacrylic acid)

MAA is another synthetic biomaterial that has been polymerized and used for use in wound healing. This biomaterial can be synthesized in the form of scaffolds, films and beads and even injectable hydrogels (Lisovsky et al., 2015). These have previously been shown to have angiogenic properties and promote skin grafting in rats and wound healing in diabetic mice (Lisovsky et al., 2016; Talior-Volodarsky et al., 2017). Most wound-healing studies using MAA have been done with MAA beads, which are made from poly(methacrylic acid-co-methyl methacrylate), containing 45% MAA, 54% methyl methacrylate, and cross-linked with 1% ethylene glycol dimethacrylate. These studies however do not investigate the effects of MAA on local chemokine signaling.

One study that examines the use of a hydroxamated version of MAA (HX-MAA) created from a two-step chemical reaction involving hydroxylamine does discuss chemokine effects. This form is composed of 65% MAA and can chelate and inactivate the zinc-containing form of matrix metalloproteinases, which authors of the study thought could improve this biomaterial’s healing properties. MMA and HX-MAA were compared to polymethyl methacrylate (PMMA), a plastic polymer used in bioengineering that distinguishes itself from MAA polymers by the methyl groups on its backbone carbon chain (Patel and Sefton, 2012). Discs containing either MAA, HX-MAA or PMMA beads were injected into a subcutaneous air pocket cavities in mice and then exudates from these wounds were recovered and evaluated with ELISA and qPCR and days one, four and ten.

The most significant effect on chemokine levels and gene expression was noted in the proinflammatory chemokine CCL7, which was significantly higher in the exudates of MAA and HX-MAA groups than PMMA. This was reflected by the increased cell infiltration seen on histology for HX-MAA and MAA treated groups. There were no major differences between MAA and HX-MAA and their effects on cytokine and chemokine expression or cellular infiltration, contrary to what authors had hypothesized. All MAA-based beads appeared to produce a greater inflammatory response than PMMA (Patel and Sefton, 2012). The authors postulate that this increased inflammation seen using MAA based materials is contributing to the advances in wound healing seen in prior studies, but no chemokines associated with anti-inflammatory or angiogenic signaling are identified in this study. The role of CCL7 as we understand it is mainly limited to binding of CCR2 expressed on macrophages to induce inflammatory cell recruitment (Saleh et al., 2022).

While research on the use of MAA biomaterials on wound healing continues, there remains little data about their effects on local chemokine signaling. Investigating this relationship will better help us understand the mechanism by which MAA helping angiogenesis. This study on HX-MAA makes MAA-based polymers appear to worsen inflammation as compared to other synthetic polymers but given that just one chemokine is examined in this study, further research is needed to elucidate their effect.

## Summary

Both natural and synthetic biomaterials have potential therapeutic uses in the field of cutaneous wound healing. The development of materials within these categories initially focused on creating polymers that mimic the natural ECM and can provide structural support and hydration to injured tissue. As we investigate the effects of these biomaterials on cellular signaling and chemokine expression, however, we learn that they play a role in wound healing that is far more complex. Among the biomaterials discussed, many appear to modulate the chemokines CCL2, CXCL8 and CXCL12 to exert downstream effects to promote wound-healing. The stimulation of pro-inflammatory and anti-inflammatory chemokine expression also appears to be highly dependent on the time frame of the biomaterial application during wound healing. Many of the discussed studies are done *in vitro* in isolation on a single cell type, which is not reflective of wound healing processes *in vivo*. Therefore, our understanding of chemokine activity in normal and pathological wound healing is also still far from complete. More research is needed to investigate the immunomodulatory effects of biomaterials on chemokine signaling, and particularly so with synthetic biomaterials.
